# Improving Bone Mineral Density Screening by Using Digital X‐Radiogrammetry Combined With Mammography

**DOI:** 10.1002/jbm4.10618

**Published:** 2022-03-21

**Authors:** Amshuman Rao, Elisabeth Elder, Jacqueline R. Center, Thach Tran, Nicholas Pocock, Grahame J. Elder

**Affiliations:** ^1^ University of Notre Dame Australia School of Medicine Sydney NSW Australia; ^2^ Westmead Breast Cancer Institute Westmead NSW Australia; ^3^ University of Sydney Sydney NSW Australia; ^4^ Bone Biology Division, Garvan Institute of Medical Research Darlinghurst NSW Australia; ^5^ St Vincent's Hospital Clinical School, UNSW Sydney NSW Australia

**Keywords:** DXA, OSTEOPOROSIS, OTHER ANALYSIS/QUANTITATION OF BONE

## Abstract

Fracture risk evaluation of postmenopausal women is suboptimal, but most women undergo screening mammography. Digital X‐radiogrammetry (DXR) determines bone mineral density (BMD) at the metacarpal shaft and can be performed on mammography equipment. This study examined correlations between DXR and dual‐energy X‐ray absorptiometry (DXA) in women undergoing mammography, to identify optimal DXR thresholds for triage to osteoporosis screening by central DXA. Postmenopausal women over age 50 years, recruited from Westmead Hospital's Breast Cancer Institute, underwent mammography, DXR and DXA. Agreements were determined using the area under the receiver operator characteristic (AUC ROC) curve and Lin's concordance correlation coefficient. Optimal DXR *T*‐scores to exclude osteoporosis by DXA were determined using the Youden's method. Of 200 women aged 64 ± 7 years (mean ± standard deviation [SD]), 82% had been diagnosed with breast cancer and 37% reported prior fracture. DXA *T*‐scores were ≤ −1 at the spine, hip or forearm in 77.5% and accorded with DXR *T*‐scores in 77%. For DXR and DXA *T*‐scores ≤ −2.5, the AUC ROC was 0.87 (95% confidence interval [CI], 0.81–0.94) at the 1/3 radius, and 0.74 (95% CI, 0.64–0.84) for hip or spine. DXR *T*‐scores > −1.98 provided a negative predictive value of 94% (range, 88%, 98%) for osteoporosis by central DXA. In response to a questionnaire, radiography staff responded that DXR added 5 minutes to patient throughput with minimal workflow impact. In the mammography setting, triaging women with a screening DXR *T*‐score < −1.98 for DXA evaluation would capture a significant proportion of at‐risk women who may not otherwise be identified and improve current low rates of osteoporosis screening. © 2022 The Authors. *JBMR Plus* published by Wiley Periodicals LLC on behalf of American Society for Bone and Mineral Research.

## Graphical abstract

Digital X‐radiogrammetry (DXR) determines bone mineral density (BMD) at the metacarpal shaft and can be performed on mammography equipment. In this study we examine correlations between DXR and dual‐energy X‐ray absorptiometry (DXA) in women undergoing mammography, and identify an optimal DXR threshold below which triaging for central DXA may capture a significant proportion of women at‐risk of facture, who would not otherwise be identified.

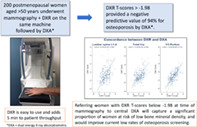

## Introduction

In 2012 it was estimated that 66% of Australians aged over 50 years had osteoporosis or osteopenia, and this number is projected to increase by 31% to 2022.^(^ ^1^ ^)^ Fragility fractures associated with reduced bone mineral density (BMD) reduce the independence and quality of life of older individuals while placing an increased financial burden on communities with an aging population. Fractures also increase premature mortality. In the first 12 months following a hip fracture, excess mortality increases from 20% to 33%, with similar increases following fractures of the pelvis and femur.^(^ ^2^ ^)^ Spine and other nonhip fractures also have an excess mortality, albeit lower than that of the hip.^(^ ^2^ ^)^

Postmenopausal women with low BMD are at increased fracture risk, but often remain undiagnosed or untreated despite the availability of treatments with proven fracture reducing efficacy.^(^ ^3^ ^)^ Currently, Australian guidelines for screening of BMD by dual‐energy X‐ray absorptiometry (DXA) provide reimbursement to people suffering a minimal trauma fracture, aged ≥70 years and when a medical condition or treatment predisposes to rapid BMD loss. Once low BMD is established, further screening is permitted at specified time intervals. Referrals for screening often follow a hospital admission or primary practitioner consultation for fracture, but evaluation of fracture risk in subjects without fracture remains slow. For women aged >65 years, only 20% to 25% currently undergo DXA examination per year, despite the great majority being eligible for a Medicare rebatable DXA examination (http://medicarestatistics.humanservices.gov.au/statistics/mbs_item.jsp).

The United States Preventive Services Task Force (USPSTF) recommends that women ≥65 years should all have screening with bone measurement testing. It also recommends that women <65 years of age should be screened with bone measurement testing if they are at an increased risk of osteoporosis, as determined by clinical risk assessment tools.^(^ ^4^ ^)^ Despite these recommendations, screening of Australian women >65 years, or <65 years with increased risk of fracture remains unacceptably low. In some communities, screening for osteoporosis may be limited by distance to a DXA facility, and cost limits accessibility when reimbursement criteria are not met. However, is often more likely that poor screening rates for osteoporosis in Australia are due in greater part to low public awareness of osteoporosis or by additional logistical difficulties for patients in arranging bone density tests.

Cancer screening programs have been established in many countries, and guidelines for breast cancer screening are generally consistent across the 21 countries with the highest per capita spending on health care.^(^ ^5^ ^)^ Annual or second yearly mammography is performed from ages 50 to 74 years in Australia, and both general screening programs and programs stratified by risk are likely to be cost effective compared to no screening.^(^ ^6^ ^)^ Between 2018 and 2019, 55% of women in the targeted age group of 50–74 years participated in the BreastScreen Australia program,^(^ ^7^ ^)^ with an additional significant proportion undergoing “defacto” private screening. These postmenopausal women are at risk of osteoporosis and provide an easily accessible population in which to implement an osteoporosis screening program. One technique trialed in this setting is digital X‐radiogrammetry (DXR), which analyses BMD from the first to fourth metacarpals of a nondominant hand radiograph to generate a BMD value, *T*‐score and *Z*‐score. DXR assessment can be performed on the same equipment used for mammography, and adding DXR to a mammogram has been reported to have little impact on work throughput.^(^ ^8^ ^)^ DXR of the nondominant hand and femoral neck DXA correlate closely, and using DXR with application of a triage model, 70% of individuals were correctly diagnosed to have or not to have a femoral neck *T*‐score ≤ −2.5, with no significant difference in discrimination between DXR and lumbar spine DXA.^(^ ^9^ ^)^

The aim of this study was to examine the correlation between DXR and DXA measured at different sites in subjects over 50 years presenting for mammography, and to identify the optimal threshold for DXR to screen for osteoporosis as diagnosed by central DXA, in this setting.

## Patients and Methods

Between August 2014 and February 2016, participants were recruited from the diagnostic imaging clinic of the Breast Cancer Institute of Westmead Hospital, which provides breast imaging to women with abnormal breast findings and to women treated for breast cancer. Participants were aged >50 years and were postmenopausal, or were ≥55 years of age with a prior hysterectomy. All patients provided informed consent.

DXR images were acquired immediately after mammography, using the same General Electric Senographe Essential DS (GE Healthcare, Cardiff, UK) equipment and an imaging preset (35 kV and 22 mAs). The nondominant hand was examined, except for participants with a hand fracture or functional injury of ≥6 months duration. Deidentified, encrypted images were forwarded to Sectra “OneScreen” and analyzed utilizing an active shape model to identify the narrowest regions of the second, third, and fourth metacarpals. The DXR report included BMD (g/cm^2^), and *T*‐scores and *Z*‐scores based on a North American white female reference population.^(^ ^10^ ^)^ All patients then underwent BMD by DXA, assessed at the forearm, both hips and spine using a Lunar Prodigy Bone Densitometer (software version 14.10; GE Lunar, Madison, WI, USA). *T*‐scores and *Z*‐scores were calculated using Geelong, Australia normative data. *Z*‐scores were adjusted for age, weight, gender, and ethnicity. The trabecular bone score (TBS) was acquired from DXA lumbar spine images and graded normal (>1.31), intermediate (1.21–1.31), or low (<1.21). Body composition was also acquired by DXA.

Participants completed a questionnaire including medical history, factors affecting BMD, falls and fracture risk, age at menopause, and prescribed and complementary medications. Radiography staff also completed a questionnaire regarding changes to workflow following the introduction of DXR testing.

Descriptive statistics and frequency tables were used for baseline characteristics and questionnaire responses. Linear regression modeling was used to determine correlations between components of the questionnaire, body composition data, TBS and DXR, and DXA *T*‐scores and BMD values.

Concordance of DXR and DXA *T*‐scores at each site was assessed using Lin's concordance correlation coefficient, where values of 1 denote perfect concordance. If DXA is considered the gold standard, then the concordance coefficient is a measure of both accuracy and consistency. Limits of agreement (LOA) were assessed using Bland‐Altman plots. Receiver operator characteristic (ROC) curve analysis was used to model relationships between DXR and DXA. Deming regression, an extension of linear regression to handle random measurement errors in DXR assuming no measurement error in DXA, was used to assess agreement of DXR and DXA. We used the Youden's method^(^ ^11^ ^)^ in conjunction with ROC curve analysis to identify the optimal threshold DXR to diagnose osteoporosis, defined as a DXA *T*‐score ≤ −2.5 at the total hip, femoral neck, or lumbar spine. In terms of optimization, the Youden's method is identical to the method that maximizes the sum of sensitivity and specificity, and to the criterion that maximizes concordance. In conjunction with the ROC analysis, it is one of the most widely used methods to identify optimal cutoff points. As the use of aromatase inhibitors might affect the agreement between DXA and DXR, we conducted a sensitivity analysis to examine the difference in concordance between DXR and DXA *T*‐scores between women with breast cancer treated with or without aromatase inhibitors. Age adjusted and multivariate logistic regression models were conducted to determine the contribution of the DXR *T*‐score to DXA‐derived osteoporosis at either the total hip, femoral neck, or lumbar spine. Based on an earlier study,^(^ ^12^ ^)^ a minimum sample size of 150 participants was calculated to provide 90% power to achieve a 5% level of significance for correlations of BMD by DXR and DXA. A target was set at 200 participants to allow for dropouts. Calculations were performed using IBM SPSS Statistics version 26 (IBM Corp., Armonk, NY, USA), SAS version 9.2 (SAS Institute Inc., Cary, NC, USA) and the Youden's index was calculated using the “ModelGood” package (https://CRAN.R-project.org/package=ModelGood). Ethical approval was granted by the Human Research Ethics Committees of Western Sydney Local Health District, and the study was registered with the Australian New Zealand Clinical Trials Registry (ACTRN12614001230640).

## Results

Of 216 women who provided informed consent, 16 failed to attend their appointment or had incomplete data, and 200 (93%) completed the study. Baseline demographics, relevant medical conditions, risks for fracture, and prescribed drugs and supplements used by the 200 women enrolled in the study are detailed in Table 1.

**Table 1:** Baseline Demographics, Relevant Medical Conditions, Risk Factors for Fracture, Supplements and Prescribed Drugs Used by Study Participants

Baseline demographics (*n* = 200)	Value
Age (years), mean ± SD	64 ± 7
BMI (kg/m^2^), mean ± SD	28.9 ± 5.7
Estimated total lean mass (%), mean ± SD	56.5 ± 8
Estimated total fat mass (%), mean ± SD	43 ± 7.5
Age at menopause (years), mean ± SD	49.8 ± 4.7
Menopause before age 50 years, *n* (%)	70 (35)
Smoking ever, *n* (%)	64 (34)
Smoking current, *n* (%)	10 (5)
Alcohol >3 standard drinks daily, *n* (%)	4 (2)
Regular physical activity, *n* (%)	136 (68)
Medical history, *n* (%)	
Diagnosed with breast cancer	164 (82)
Treated with chemotherapy	68 (34)
Endocrine therapy: aromatase inhibitor	48 (24)
Endocrine therapy: tamoxifen	30 (15)
Diabetes mellitus type 2	26 (13)
Diabetes mellitus type 1	2 (1)
Rheumatoid arthritis	12 (6)
Hyperthyroidism	4 (2)
Anorexia nervosa	1 (0.5)
Bulimia	2 (1)
Premenopausal amenorrhea >3 months	20 (10)
Fracture risks, *n* (%)	
Falls in the last 12 months	24 (12)
Impaired balance	16 (8)
Impaired vision	12 (6)
Decrease in height of >1.5 cm	14 (7)
Prior fracture	74 (37)
Family history of parental hip fracture	8 (4)
Self‐stated diagnosis of osteoporosis	36 (18)
Drugs and supplements, *n* (%)	
Dietary calcium intake low versus intermediate or high	64 (32)
Supplemental calcium	88 (44)
Cholecalciferol	120 (60)
Menopausal hormonal therapy	56 (28)
Current bisphosphonates	24 (12)
Glucocorticoids >3 months	38 (19)

BMI = body mass index; SD = standard deviation.

The mean age of the 200 women was (mean ± standard deviation [SD]) 64 ± 7 years, and 163 (82%) had a diagnosis of breast cancer with a median period since diagnosis of 3.7 years (interquartile range [IQR], 1.9–7.2). Menopausal hormonal therapy had been used by 28% of women with and 28% without a diagnosis of breast cancer.

Radiography staff answered their questionnaire at the end of the study recruitment. They indicated that DXR after mammography was convenient, added approximately 5 minutes to the throughput of each patient and had a minimal impact on workflow. No harm was associated with this study.

By DXA, 157 (78.5%) of participants had a *T*‐score ≤ −1 at any spine, hip, or forearm site, whereas 61 (30.5%) had a *T*‐score ≤ −2.5 at any site (Table 2). At the femoral neck, total hip, or spine, the sites most often used for a diagnosis of osteoporosis or osteopenia, 130 (65%) of participating women had a *T*‐score ≤ −1, of whom 28 (14% of the total) had a *T*‐score ≤ −2.5 in the osteoporotic range. For 72 participants under 60 years of age, 35 (48.6%) had *T*‐scores at the hip or spine in the osteopenic range whereas 6 (8.3%) had *T*‐scores in the osteoporotic range.

**Table 2:** DXR and DXA AUC ROC by DXA Site and *T*‐Score Range, and Number of Participating Women in Each DXA‐Derived *T*‐Score Category

DXA‐derived *T*‐score^a^	DXA site/sites used to generate *T*‐score	Women with osteoporosis/osteopenia *n* (%)	ROC AUC (95% CI) for DXR and DXA, within the DXA *T*‐score range
≤ −1	Hip, spine, or forearm	157 (78.5)	0.85 (0.79–0.91)
≤ −2.5	Hip, spine, or forearm	61 (30.5)	0.79 (0.73–0.86)
≤ −1	Hip or spine	130 (65.0)	0.75 (0.67–0.82)
≤ −2.5	Hip or spine	28 (14.0)	0.74 (0.64–0.84).
≤ −2.5	1/3 radius	21 (10.5)	0.87 (0.81–0.94)

AUC = area under the ROC curve; CI = confidence interval; DXA = dual‐energy X‐ray absorptiometry; DXR = digital X‐radiogrammetry; ROC = receiver operator characteristic.

^a^ DXA‐derived T‐score range ≤ −1 or ≤ −2.5.

For the 128 participants aged 60 and over, 66 (51.6%) had *T*‐scores in the osteopenic range and 22 (17.2%) in the osteoporotic range. By DXR, a similar number of women (132 women; 66%) had a *T*‐score ≤ −1, but almost twice as many (52 women; 26%) had a *T*‐score ≤ −2.5 by DXR compared to DXA.

**Fig. 1:**
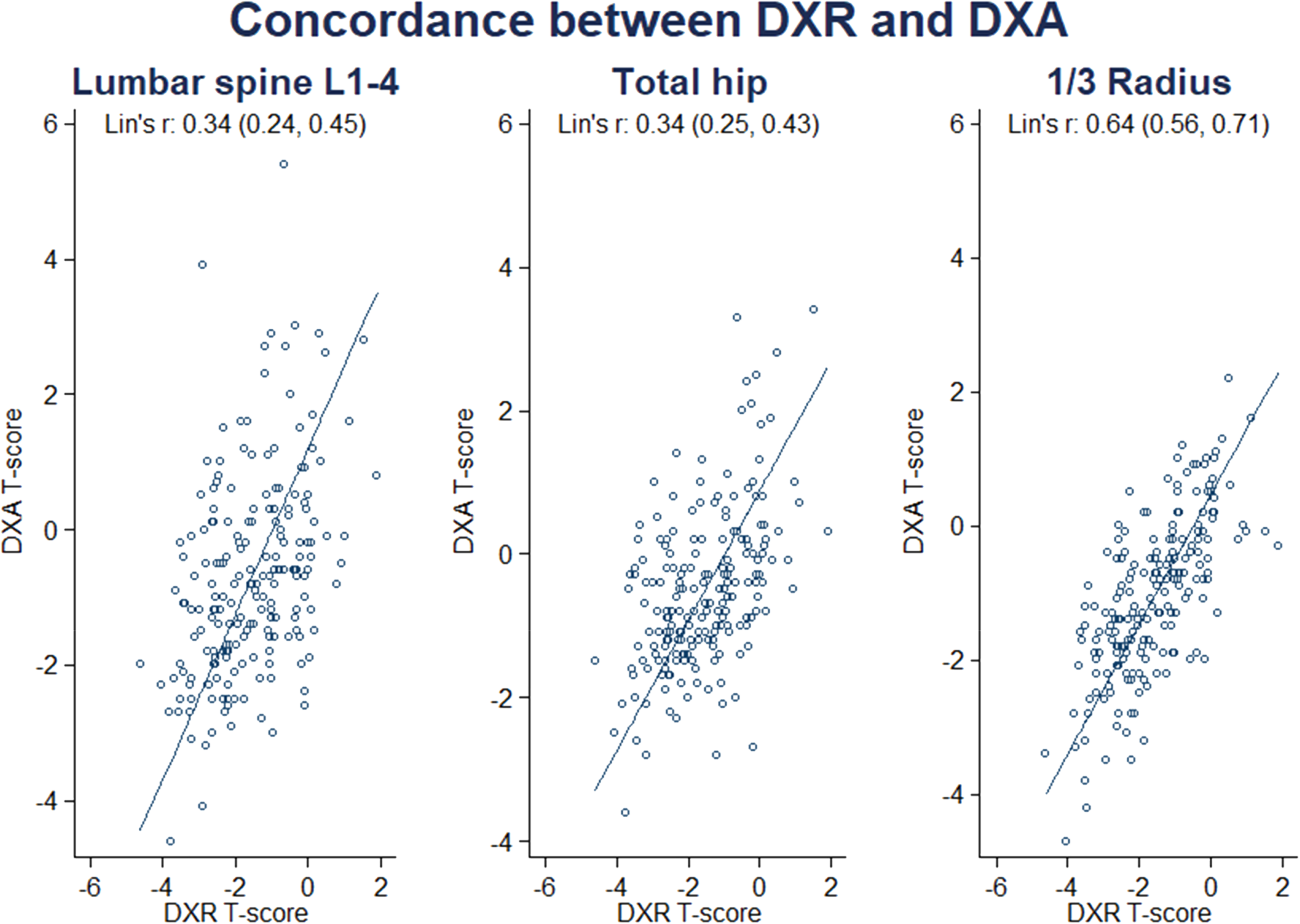
Concordance between DXR and DXA at the lumbar spine, total hip, and 1/3 radius.

**Fig. 2:**
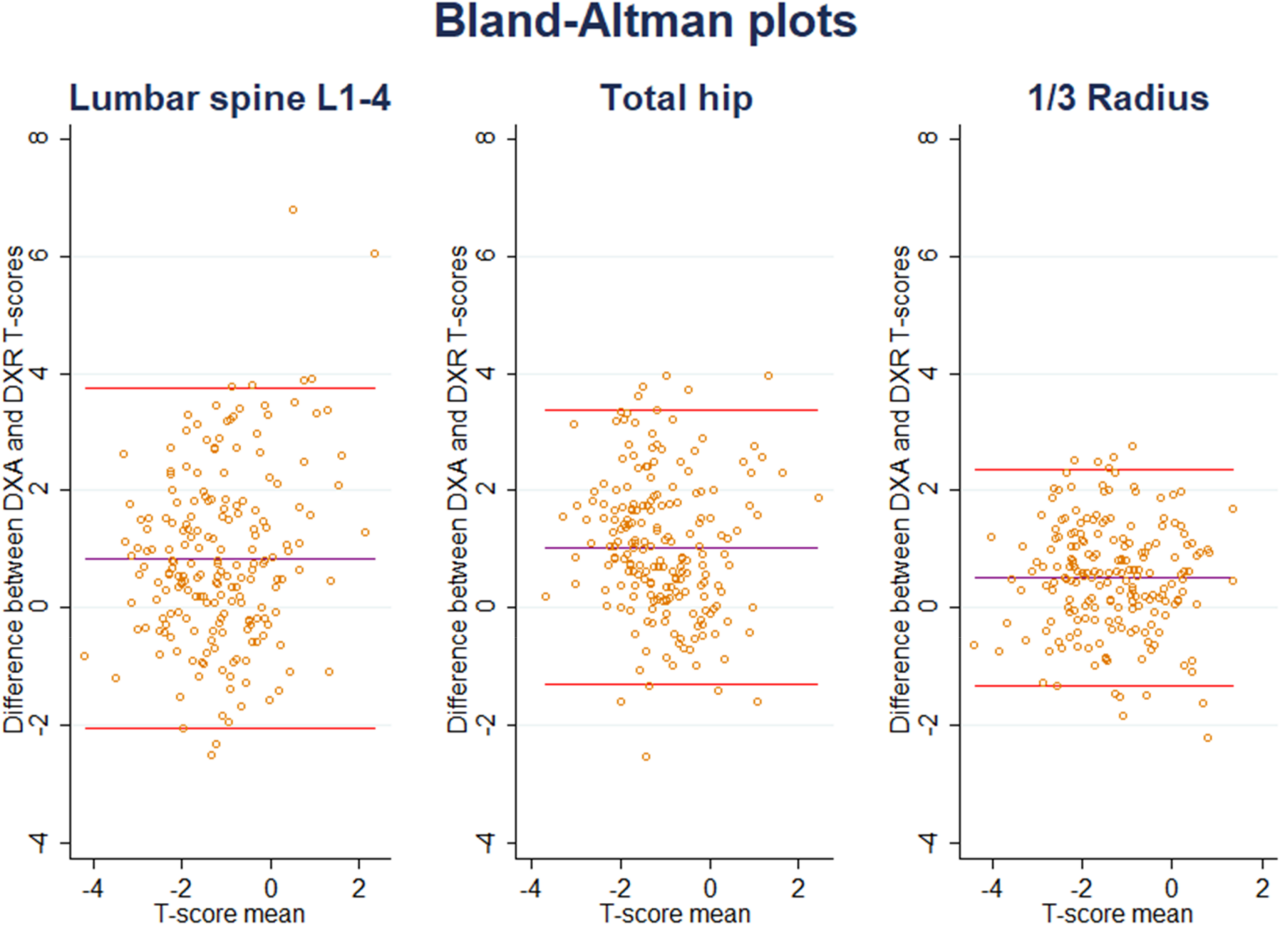
Bland‐Altman plots for the lumbar spine, total hip, and 1/3 radius.

DXR measures BMD at the metacarpal shaft, which is exclusively cortical bone. DXR *T*‐scores correlated for all DXA sites, but most closely at the distal 1/3 radius, the site with the highest proportion of cortical bone (Table 3). Concordance of *T*‐scores derived by DXR and DXA by site is indicated in Fig. 1. Lin's concordance correlation coefficient between DXR and DXA at the spine or hip was low, but there was a reasonable concordance coefficient between DXR and the one‐third radius by DXA, with Lin's concordance coefficient 0.64 (95% confidence interval [CI], 0.56–0.71; *p* < 0.001) and 95% LOA assessed using Bland‐Altman plots of −1.32 to 2.35 (Fig. 2). The sensitivity and specificity of DXR for a DXA‐derived diagnosis of osteoporosis using the area under the receiver operator characteristic (AUC ROC) curve ranged from 0.74 for DXA derived *T*‐score ≤ −2.5 at the hip or spine, 0.79 for *T*‐score ≤ −2.5 by DXA at hip, spine or forearm, and 0.87 if the DXA 1/3 radius was used (Table 2). For DXR and a DXA *T*‐score of ≤ −2.5 at any site, the AUC was 0.79 (95% CI, 0.73–0.86) and was 0.76, 0.77, and 0.80 for respective age ranges of 50–59, 60–69, and ≥70 years. For DXR and DXA *T*‐scores ≤ −2.5 at the 1/3 radius, the AUC was 0.87 irrespective of age.

**Table 3:** Correlations of *T*‐Scores Derived Using DXA and DXR by Site

DXA and DXR	Mean *T*‐score (mean ± SD)	DXR and DXA *T*‐score correlation (all *p* < 0.001)
DXA		
Lumbar spine	−0.72 ± 1.49	0.418
Total proximal femur	−0.53 ± 1.11	0.478
Femoral neck	−0.83 ± 1.03	0.486
1/3 Radius	−1.05 ± 1.18	0.695
DXR	−1.57 ± 1.22	–

DXA = dual‐energy X‐ray absorptiometry; DXR = digital X‐radiogrammetry.

The mean TBS (L_1_–L_4_) was in the low normal range at 1.31 ± 0.13. The TBS correlated significantly to DXA *T*‐scores at all sites, but most closely at the lumbar spine (0.265; *p* < 0.001) and the total radius (0.221; *p* = 0.002). The TBS and DXR *T*‐scores correlated poorly (0.143; *p* = 0.046).

Deming regression was used to quantify the correlation between the two diagnostic methods. For a DXA *T*‐score ≤ −2.5 at the lumbar spine, femoral neck, or total hip, the corresponding DXR *T*‐score was −3.05 (DXA *T*‐score = 0.005 + 0.82 × DXR *T*‐score). Although DXR *T*‐score thresholds for DXA *T*‐scores ≤ −2.5 were similar at all sites, they were most similar at the forearm, being −3.09 for DXA *T*‐scores ≤ −2.5 at the 1/3 radius.

### Selection of the optimal threshold for DXR to diagnose osteoporosis

We found a DXR value of −1.98 was associated with the highest Youden's index, suggesting that this value was the optimal DXR threshold below which to screen for osteoporosis at any of the spine, total hip, or femoral neck DXA sites. Similarly, women with DXR values > −1.98 would have a high probability of not having osteoporosis; ie, a negative predictive value of 94% (range, 88%, 98%) by central DXA. Exploratory analyses in Table 4 indicate different DXR *T*‐score thresholds to diagnose osteoporosis at individual DXA sites. If the DXR threshold of −1.98 is used, 117 subjects (~58.5% of the whole study sample) would not need a DXA following a DXR test, and 83 subjects (~41.5% of the sample) would be referred to test DXA. Using this threshold, we would be able to capture as many as 21 of 28 or 75% of all DXA‐confirmed osteoporotic subjects. Corresponding diagnostic parameters of this threshold are provided in Table 4.

**Table 4:** Predictive Performance for Different DXR *T*‐Score Thresholds to Diagnose Osteoporosis Operationally Defined Using DXA *T*‐Scores at Different Sites

DXA *T*‐score site	Total hip, femoral neck or lumbar spine	Total hip	Femoral neck	Lumbar spine	1/3 Radius
Prevalence of osteoporosis using DXA, % (95% CI)	14.0 (9.5, 19.6)	3.0 (1.1, 6.4)	3.0 (1.1, 6.4)	11.0 (7.0, 16.2)	10.5 (6.6, 15.6)
DXR *T*‐score threshold	−1.98	−3.18	−2.50	−1.98	−1.81
Sensitivity, % (95% CI)	75.0 (55.1, 89.3)	66.7 (22.3, 95.7)	66.7 (22.3, 95.7)	81.8 (59.7, 94.8)	100 (93.9, 100)
Specificity, % (95% CI)	64 (56.3, 71.1)	90.7 (85.7, 94.4)	75.3 (68.6, 81.2)	63.5 (56.0, 70.6)	60.9 (53.3, 68.1)
AUC (95% CI)	0.70 (0.61, 0.78)	0.79 (0.58, 0.99)	0.71 (0.50, 0.92)	0.73 (0.64, 0.81)	0.80 (0.77, 0.84)
Positive likelihood ratio (95% CI)	2.08 (1.55, 2.79)	7.19 (3.51, 14.7)	2.69 (1.45, 4.99)	2.24 (1.70, 2.95)	2.56 (2.13, 3.07)
Negative likelihood ratio (95% CI)	0.39 (0.20, 0.75)	0.37 (0.12, 1.14)	0.44 (0.14, 1.38)	0.29 (0.12, 0.70)	0
Positive predictive value, % (95% CI)	25.3 (16.4, 36.0)	18.2 (5.2, 40.3)	7.7 (2.1, 18.5)	21.7 (13.4, 32.1)	23.1 (14.9, 33.1)
Negative predictive value, % (95% CI)	94.0 (88.1, 97.6)	98.9 (96.0, 99.9)	98.6 (95.2, 99.8)	96.6 (91.5, 99.1)	100 (96.7, 100)

AUC = area under the curve; CI = confidence interval; DXA = dual‐energy X‐ray absorptiometry; DXR = digital X‐radiogrammetry.

Alternative statistical approaches have been suggested to determine triage thresholds for peripheral X‐ray absorptiometry devices. The “equivalent *T*‐score” uses upper and lower triage thresholds, with the upper threshold defined by Blake and colleagues^(^ ^13^ ^)^ as the peripheral *T*‐score below which 90% of the osteoporotic women lay, and the lower threshold as the *T*‐score above which 90% of the non‐osteoporotic women lay. Because this approach has been widely used in peripheral DXA (pDXA) research,^(^ ^14^ ^)^ we performed an analysis using both the upper and lower triage and Youden methods. Neither the upper nor the lower triage threshold was practically better than the Youden's threshold if all practical implication issues were included (ie, the number of women referred to DXA scan following a DXR scan or the number. of osteoporotic women who would have been undiagnosed). Results of these analyses are included in Supplementary Table S1.

The performance of the Youden‐derived threshold was next tested by splitting the original cohort randomly 150:50 into a development and validation set. The analysis using the development cohort suggested an optimal DXR threshold of −1.93, which was indeed very close to the threshold of −1.98 reported in the primary analysis. This threshold was then validated in the validation cohort of 50 subjects. The results of the validation analysis were consistent with that in the development cohort as well as the primary analysis results, confirming the robustness of the primary analysis (Supplementary Table 2).

### Contribution of DXR *T*‐scores to risk of osteoporosis and fracture

We next assessed the contribution of the DXR *T*‐score and other variables derived from the patient questionnaire to the risk of osteoporosis diagnosed by central DXA (Table 5). The analysis indicated that for every one DXR *T*‐score reduction there was a twofold increased risk of osteoporosis defined by the lowest *T*‐scores at either total hip, femoral neck, or lumbar spine. The contribution of DXR to the risk of osteoporosis remained significant even after accounting for potential confounding effects. A sensitivity analysis was also performed to assess effects of aromatase inhibitors on the agreement between DXA and DXR. The analysis suggested that women with breast cancer treated and treated with aromatase inhibitors had higher DXR and DXA *T*‐scores at the total hip, including after accounting for the difference in age at entry between the two groups (Supplementary Table 3). Additionally, there was a trend toward poorer concordance between DXR and DXA *T*‐scores in women with breast cancer treated with aromatase inhibitors, although the numbers in this group were small (Fig. 1). A larger study is warranted to confirm this exploratory finding. Using DXA or DXR *T*‐scores in Fracture Risk Assessment Tool (FRAX®) for prediction of any osteoporotic fracture within 10 years, Lin's correlation coefficient Rho c was 0.680 (95% CI, 0.615–0.744) with 98% agreement, and using the Garvan fracture risk calculator, Lin's concordance correlation coefficient for any fracture within 10 years and all ages was 0.767 (95% CI, 0.719–0.815) with 81% agreement.

**Table 5:** Contribution of DXR *T*‐score and Other Variables to the Risk of Osteoporosis Diagnosed by Central DXA

Variable	Unit	Age‐adjusted OR (95% CI)	Multivariable‐adjusted OR (95% CI)
DXR *T*‐score	1 *T*‐score decrease	**2.30 (1.46, 3.62)**	**2.11 (1.30, 3.43)**
Age (years)	1 year increase		1.01 (0.93, 1.10)
BMI (kg/m^2^)	1 kg/m^2^ decrease	**1.16 (1.05, 1.27)**	**1.14 (1.03, 1.26)**
History of prior fracture	Yes	1.11 (0.48, 2.59)	0.85 (0.31, 2.30)
Self‐stated diagnosis of osteoporosis	Yes	2.89 (1.17, 7.13)	2.36 (0.82, 6.82)
History of falls in the last 12 months	Yes	0.46 (0.10, 2.13)	0.76 (0.14, 4.05)
Use of calcium supplements	Yes	1.18 (0.49, 2.87)	1.14 (0.41, 3.16)
Use of vitamin D supplements	Yes	1.16 (0.50, 2.70)	1.12 (0.42, 2.98)
Use of postmenopausal hormone therapy	Yes	0.45 (0.16, 1.28)	0.68 (0.22, 2.11)
Use of corticosteroid	Yes	0.93 (0.33, 2.67)	0.96 (0.31, 3.04)
Family history of parental hip fracture	Yes	1.99 (0.37, 10.7)	2.33 (0.33, 16.37)

Data presented as ORs (95% CI). Bold font indicates statistical significance.

BMI = body mass index; CI = confidence interval; DXA = dual‐energy X‐ray absorptiometry; DXR = digital X‐radiogrammetry; OR = odds ratio.

## Discussion

In this study, DXR correlated well with DXA as has been noted in earlier studies. However, we also establish a DXR *T*‐score < −1.98 as the optimal threshold for DXR to screen for DXA defined osteoporosis in subjects presenting for mammography. Although some women with a DXR *T*‐score < −1.98 would be incorrectly classified to have osteoporosis, those women would be identified if that DXR *T*‐score threshold was the basis for proceeding to DXA evaluation. By corollary, DXR *T*‐scores > −1.98 indicated a relatively low risk of DXA osteoporosis, with a negative predictive value of 94% (range, 88%, 98%), and women in that group were unlikely to have osteoporosis at the spine or hip by DXA. Nevertheless, the major concern for a DXR screening program is that some women with a DXR *T*‐score ≥ −1.98 may be misclassified to have a BMD above the osteoporotic range, despite having osteoporosis at the hip or spine by DXA. However, for women assessed at low risk by DXR, existing guidelines for referral to DXA based on age, medication use, prior fragility fracture, or hormonal conditions predisposing to osteoporosis would remain applicable, and those women would not be lost as “false negatives.” Hence, using a DXR threshold for triaging to DXA may identify a previously unrecognized and at‐risk population, without jeopardizing the identification of osteoporosis in women meeting usual screening criteria.

A DXR value of −1.98 was associated with the highest Youden's index, suggesting this was the optimal DXR cutoff point, below which to screen for osteoporosis by DXA. Although Youden's criterion is the most common method used to identify the threshold for diagnostic testing, because it is able to maximize both sensitivity and specificity, other methods have been suggested. We therefore performed an analysis suggested for pDXA to determine the upper triage threshold *T*‐scores, but neither the lower nor the upper triage threshold was practically better than the Youden's threshold.

DXR measures BMD at the metacarpal shafts, which are exclusively cortical bone, and therefore similar to the 1/3 radius shaft. BMD by DXR correlated with BMD by DXA at all sites, but most closely at forearm sites, with Lin's concordance coefficient for DXR and DXA at the distal 1/3 radius 0.64 (95% CI, 0.56–0.71; *p* < 0.001). Despite concordance being lower at the hip and spine, the AUC ROC curve for DXR and a diagnosis by DXA of osteoporosis at the hip or spine was acceptable at 0.74 (95% CI, 0.64–0.84) and for a DXA *T*‐score of ≤ −2.5 at any site the AUC was 0.79 (95% CI, 0.73–0.86).

Women in this study had numerous risk factors for low BMD. All participants were postmenopausal, with a mean age at menopause of 49.8 ± 4.7 years, and age at the time of examination was 64 ± 7 years. The majority had a diagnosis of breast cancer, many of whom were treated with chemotherapy or medications to induce estrogen deficiency and 37% reported a prior fracture. Of the 200 women, 14% were found to have a *T*‐score ≤ −2.5 at the hip or spine by DXA, and 50.5% of women in the study were osteopenic at the hip or spine. For the 72 women under age 60 years, rates of osteoporosis and osteopenia based on DXA at the hip and spine were 8.3% and 48.6% respectively, which fell within the expected range for an Australian population of similar age.^(^ ^15^ ^)^ For 128 women aged 60 years and over, osteopenia was present in 51.6%, which is within the expected range of 51.4% to 48% for Australian women aged 60–69 years. Only 17.2% of these older women had DXA hip or spine *T*‐scores in the osteoporotic range, despite apparent increased risk factors, which compares to 21% to 24% with osteoporosis in a similarly aged Australian population study that recruited participants from 1994 to 2006.^(^ ^15^ ^)^ In that study, the frequency of antiresorptive medication use, menopausal hormonal therapy (MHT) and differences in body mass index (BMI), among other factors, may have contributed to a higher rate of osteoporosis than the current study.

Several organizations support screening to detect osteoporosis for women aged ≥65 years, and for postmenopausal women age <65 years with additional risk factors. These include the National Osteoporosis Foundation,^(^ ^16^ ^)^ the International Society for Clinical Densitometry (ISCD; https://www.aub.edu.lb/fm/CaMOP/Documents/iscd-adult-official-positions.pdf), and the U.S. Preventative Services Task Force (USPSTF). The USPST concluded there was a net benefit for osteoporosis screening of postmenopausal women aged ≥65 years, with moderate evidence for the value of screening women <65 years who are at increased risk of osteoporosis (https://www.uspreventiveservicestaskforce.org/uspstf/recommendation/osteoporosis‐screening). Despite these recommendations, no country has instigated population‐based screening from age 65 years.

A recent large‐scale study of DXR at the time of general mammography screening (without DXA correlation) reported that DXR *T*‐scores predicted hip, major osteoporotic fracture (MOF), and clinical fractures over a median follow‐up of 3.3 years^(^ ^17^ ^)^ with AUC of 0.79, 0.69, and 0.65, respectively. However, in models that included age and other clinical risk factors, the statistically significant association between DXR *T*‐scores and hip fractures was mitigated in this relatively young cohort. An earlier study reported that for prediction of MOF and hip fracture, the AUC was similar for femoral neck BMD (0.68 and 0.75, respectively), lumbar spine BMD (0.65 and 0.69, respectively), DXR‐BMD (0.65 and 0.69, respectively) and FRAX (0.64 and 0.70, respectively).^(^ ^18^ ^)^ In 2008 the ISCD published a position statement, which included comment on the use of pDXA and validating new equipment.^(^ ^14^ ^)^ DXR was specifically excluded from the statement. However, as a rule, new devices require longitudinal study with fracture outcomes for validation against existing instruments. Cross‐sectional studies may be acceptable if there is close correlation (>0.8) with a validated device, good standardization precision, and similar discrimination between fractured and not fractured age‐matched controls. Although this approach is necessary for devices that may replace validated instruments, the current study used DXR to triage women at greatest risk to DXA, rather than to replace DXA.

As shown in this study, almost two‐thirds of postmenopausal women presenting for mammography have osteopenia or osteoporosis by DXA, and screening with DXR at the time of mammography has some advantages over the aforementioned strategies. Mammography and concurrent DXR can be performed in existing clinical settings in a quick, convenient, and safe way, and DXR has potential for incorporation into established screening programs. In this study, radiographers found that DXR after mammography was convenient and added approximately 5 minutes to patient throughput. Therefore, a model of care where all subjects aged 50–74 years presenting for mammography also underwent screening DXR would allow a significant proportion of at‐risk individuals to be identified and referred for additional DXA assessment, with minimal interruption to workflow.

The current Australian national breast cancer screening program reaches the majority of women aged 50–74 years, and the combination of mammography and osteoporosis screening using DXR may provide convenient and improved access to densitometry services in some areas. For patients diagnosed with breast cancer and treated with estrogen deprivation therapies, DXR may also be a useful screening tool to identify women who are more likely to require interventions to reduce BMD loss. If DXR *T*‐scores were < −1.98, then further assessment by DXA could be undertaken. However, alternative thresholds may be useful for particular patient groups. For example, women treated with aromatase inhibitors are more prone to loss of BMD, and for those women, a higher DXR threshold might be appropriate for referral for BMD assessment by DXA. Nevertheless, in the current study the selected DXR threshold appears appropriate to women on aromatase inhibitors, because they were not found to have lower BMD at any site.

This study has some limitations. Women in the study differ from the general population undergoing mammographic screening because 82% had a diagnosis of breast cancer and some were on medications that may have reduced BMD. However, the prevalence of osteopenia and osteoporosis was within the expected population range, except for women in the study aged 60 years and over who had a lower prevalence of osteoporosis. Nevertheless, their demographics (Table 1) are similar to women of similar age in the general community. The mean age of women in this study was 64 ± 7 years and they were predominantly white, so these data may not be applicable to younger women or to other ethnic groups. The normative populations used for DXA and DXR *Z*‐score and *T*‐score generation differed, so that *T*‐scores for an individual would be expected to differ for DXA and DXR. Nevertheless, both DXR and DXA *T*‐scores have been shown to predict fracture outcomes in diverse populations. We also acknowledge that most fractures occur in women with BMD in the osteopenic range, and a DXR *T*‐score > −1.98 does not exclude fracture risk despite a low risk for osteoporosis by DXA. However, DXR in combination with mammography could be used to identify women with a lower relative fracture risk and may identify women at increased relative risk in need of confirmatory DXA, who may not otherwise have undergone BMD screening. Most treatment algorithms currently include increased absolute fracture risk (AFR), as assessed either by the Garvan Fracture risk calculator (https://www.garvan.org.au/bone-fracture-risk) or by FRAX (https://www.sheffield.ac.uk/FRAX/index.aspx) as an indication for therapeutic intervention. Results from DXR are not established for calculation of AFR using these algorithms. Therefore, the current study cannot exclude that some subjects with DXR *T*‐score > −1.98 may have increased absolute fracture risk. Finally, we did not conduct a cost benefit analysis, and rather assessed whether DXR could be integrated into a busy mammography program.

This study shows that almost two‐thirds of postmenopausal women presenting for follow‐up mammography have osteopenia or osteoporosis by DXA. Few of these participants had undergone clinical DXA prior to enrolling in the study. For these women, BMD by DXR and DXA correlated significantly at all DXA sites, and above a DXR *T*‐score cut point of > −1.98, approximately 94% women would not have osteoporosis at central DXA sites. Using a threshold DXR *T*‐score for triaging to DXA, a model of care where screening DXR was combined with mammography would identify a significant proportion of women at risk of osteoporosis who would not otherwise have been recognized. This strategy could significantly improve the current unacceptably low rates of osteoporosis screening in Australia.

## Conflict of Interest

AR, EE, and TT have nothing to disclose. JRC declares Bayer and Amgen advisory board membership, and Amgen speaker's fee and research funding. GJE declares travel and speakers' fees from Takeda Pharmaceuticals, and a speaker's fee from Vifor Pharma Pty Ltd. NP declares consulting, speaker's fees, and research grants from Amgen and is director of Chatswood Densitometry.

## Data Availability

The data that support the findings of this study are available on request from the corresponding author. The data are not publicly available due to privacy or ethical restrictions.

### Peer Review

The peer review history for this article is available at https://publons.com/publon/10.1002/jbm4.10618.

## Supporting information


**Supplementary Table S1** Comparison of the upper and lower triage thresholds and Youden's method^(^ ^11^ ^)^ to identify the threshold for DXA to diagnose osteoporosis, defined as a *T*‐score ≤ −2.5 at the total hip, femoral neck, or lumbar spine.
**Supplementary Table S2** An exploratory analysis to test the performance of the derived threshold, with the original cohort randomly split 150:50 for a development and validation set.
**Supplementary Table S3** Difference between DXR and DXA *T*‐scores for women with breast cancer who were and were not treated with aromatase inhibitors, including after age‐adjustment. 95% CI = 95% confidence interval.
**Supplementary Fig. S1** Concordance in DXR and DXA *T*‐scores between women with breast cancer who were or were not treated with aromatase inhibitors
