# Supplementary material for: Improving Bone Mineral Density Screening by Using Digital X‐Radiogrammetry Combined With Mammography
Source: JBMR Plus. 2022 Mar 21;6(5):e10618. doi: 10.1002/jbm4.10618 (PMC9059473; doi:10.1002/jbm4.10618)

Supplementary Table 1.

	**Lower triage**	**Upper triage**	**Youden**	**Convenient**	**Maximal predictive**
DXR T-scores	-2.87	-0.95	-1.98	-2.00	-3.68
No. of non-osteoporotic women above the threshold	**155 (90.12%)**	62 (36.05%)	110 (63.95%)	111 (64.53%)	170 (98.84%)
No. of osteoporotic women below the threshold	11 (39.29%)	**25 (89.26%)**	21 (75.0%)	21 (75.0%)	3 (10.71%)
Sensitivity	60.70%	89.30%	75.00%	75.00%	10.70%
Specificity	9.88%	36%	64.00%	64.50%	98.80%
AUC	0.353	0.627	0.695	0.698	0.548
Positive predictive	9.88%	18.50%	25.30%	25.60%	60.00%
Negative predictive	60.70%	95.40%	94.00%	94.10%	87.20%
No. of women referred to DXA (below the threshold)	28 (14.0%)	135 (67.5%)	83 (41.5%)	82 (41.0%)	5 (2.5%)
No. of osteoporotic women undiagnosed	17 (60.71%)	3 (10.71%)	7 (25.0%)	7 (25.0%)	25 (89.29%)

Supplementary Table 2.

	**Development cohort (n= 150)**	**Validation cohort (n= 50)**
DXR T-scores	-1.93	
Number of osteoporotic women (DXA)	21	7
Prevalence of osteoporosis (95% CI)	14.0% (8.9%, 20.6%)	14.0% (5.8%, 26.7%)
Sensitivity	76.2% (52.8%, 91.8%)	71.4% (29.0%, 96.3%)
Specificity	63.6% (54.6%, 71.9%)	65.1% (49.1%, 79.0%)
AUC	0.70 (0.60, 0.80)	0.68 (0.49, 0.88)
Positive predictive	25.4% (15.3%, 37.9%)	25.0% (8.7%, 49.1%)
Negative predictive	94.3% (87.1%, 98.1%)	93.3% (77.9%, 99.2%)

Supplementary Table 3.

	Women with breast cancer treated with aromatase inhibitors (n= 48)	Women with breast cancer not treated with aromatase inhibitors (n= 115)	Difference (95% CI)	Age-adjusted difference (95% CI)
DXR T-scores	-1.45 (1.33)	-1.77 (1.16)	0.32 (-0.10, 0.73)	0.22 (-0.16, 0.60)
DXA T-scores				
Lumbar spine	-0.46 (1.65)	-0.86 (1.45)	0.40 (-0.11, 0.91)	0.40 (-0.11, 0.92)
Total hip	-0.12 (1.16)	-0.66 (1.08)	**0.54 (0.16, 0.91)**	**0.48 (0.11, 0.85)**
1/3 Radius	-0.91 (1.07)	-1.19 (1.28)	0.28 (-0.13, 0.70)	0.19 (-0.20, 0.59)

Supplementary Figure 1.

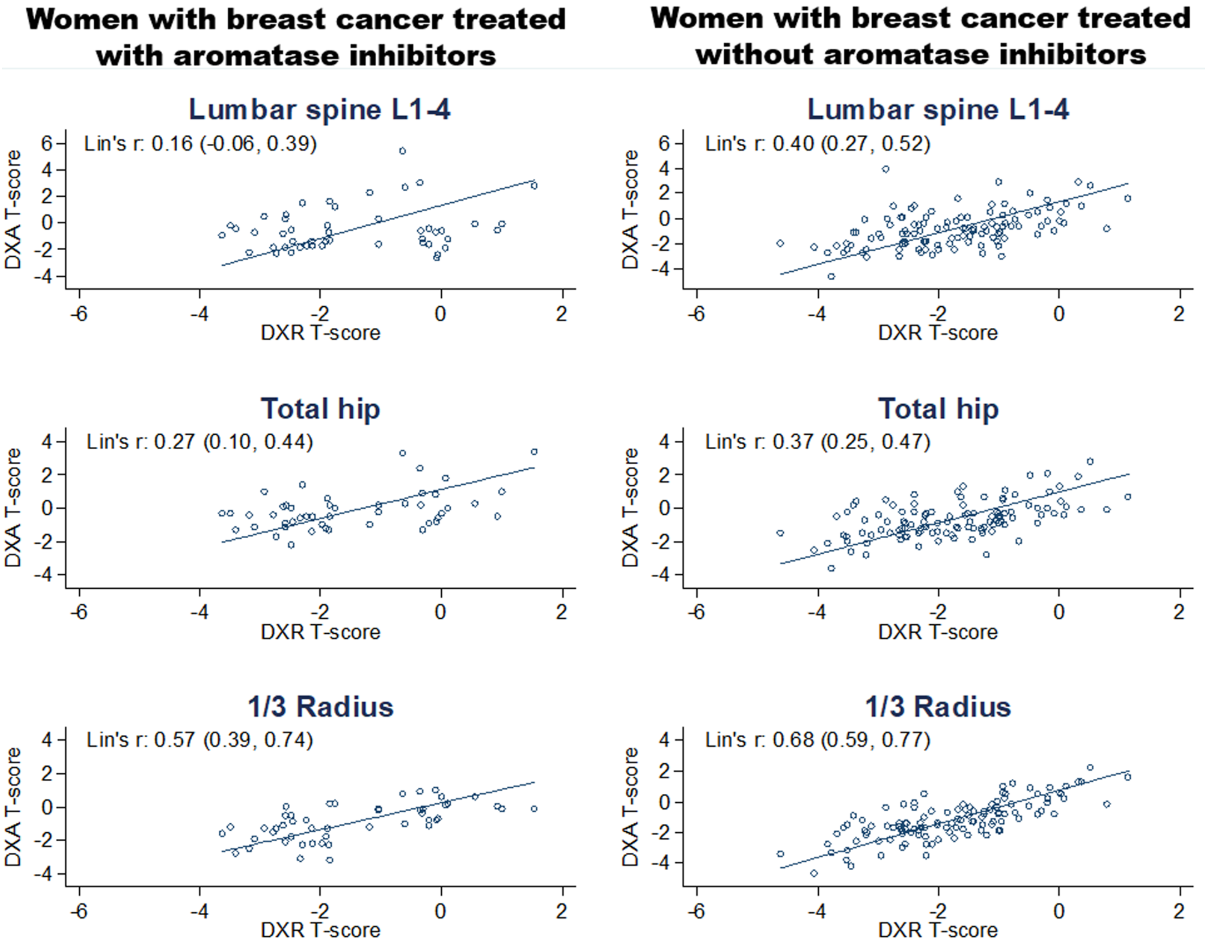

Supplement: Supplementary file 1 — Supplementary Table S1 Comparison of the upper and lower triage thresholds and Youden's method( 11 ) to identify the threshold for DXA to diagnose osteoporosis, defined as a T‐score ≤ −2.5 at the total hip, femoral neck, or lumbar spine. Supplementary Table S2 An exploratory analysis to test the performance of the derived threshold, with the original cohort randomly split 150:50 for a development and validation set. Supplementary Table S3 Difference between DXR and DXA T‐scores for women with breast cancer who were and were not treated with aromatase inhibitors, including after age‐adjustment. 95% CI = 95% confidence interval. Supplementary Fig. S1 Concordance in DXR and DXA T‐scores between women with breast cancer who were or were not treated with aromatase inhibitors [file JBM4-6-e10618-s001.pdf]
